# The Radon transform as a tool for 3D reciprocal-space mapping of epitaxial microcrystals

**DOI:** 10.1107/S1600576722004885

**Published:** 2022-07-05

**Authors:** Mojmír Meduňa, Fabio Isa, Franco Bressan, Hans von Känel

**Affiliations:** a Department of Condensed Matter Physics, Masaryk University, Kotlářská 2, CZ-61137 Brno, Czech Republic; b Laboratory for Solid State Physics, ETH Zurich, Otto-Stern Weg 1, CH-8093 Zurich, Switzerland; c G-ray Switzerland SA, Rouges-Terres 61, CH-2068 Hauterive, Switzerland; Ecole National Supérieure des Mines, Saint-Etienne, France

**Keywords:** Radon transform, X-ray diffraction, patterned Si substrates, Ge microcrystals, reciprocal-space mapping

## Abstract

This work presents a new approach suitable for mapping reciprocal space in three dimensions with standard laboratory equipment and a typical X-ray diffraction setup. The method is based on symmetric and coplanar high-resolution X-ray diffraction, ideally realized using 2D X-ray pixel detectors. The processing of experimental data exploits the Radon transform commonly used in medical and materials science. It is shown that this technique can also be used for diffraction mapping in reciprocal space even if a highly collimated beam is not available. The application of the method is demonstrated for various types of epitaxial microcrystals on Si substrates. These comprise partially fused SiGe microcrystals that are tens of micrometres high, multiple-quantum-well structures grown on SiGe microcrystals and pyramid-shaped GaAs/Ge microcrystals on top of Si micropillars.

## Abstract 2

Different types of epitaxially grown microcrystals on Si are used for the demonstration of 3D reciprocal-space map reconstruction using the Radon transform. With this technique, high-resolution diffraction reciprocal-space maps in three dimensions can be collected efficiently even with a standard laboratory setup.

## 1. Introduction

X-ray scattering measurements applied in a variety of techniques and for different samples are often realized by collecting the scattered intensity for various orientations of the incident beam and the recording detector. We recall that the intensity is mapped within reciprocal space using characteristic X-ray wavelengths and incidence and exit angles related to the sample surface (Fewster, 1994 ▸; Pietsch *et al.*, 2004 ▸; Warren, 1990 ▸). Reciprocal-space maps (RSMs) related to the sample surface are therefore constructed in this case.

There are a vast number of techniques in which reciprocal-space mapping can be realized. For instance, non-specular X-ray reflectivity (Holý *et al.*, 1993 ▸, 1993 ▸; Holý & Baumbach, 1994 ▸; Colombi *et al.*, 2008 ▸; Falub *et al.*, 2017 ▸) can be used for the detection of layer thickness and roughness, grazing-incidence small-angle X-ray scattering (GISAXS) (Levine *et al.*, 1989 ▸; Schmidbauer *et al.*, 1998 ▸) can be applied for morphological studies of very thin layers and nano­structures, small-angle X-ray scattering (Glatter & Kratky, 1982 ▸) is usually used for structural studies of nanoparticles in a bulk medium, and high-resolution X-ray diffraction (HRXRD) (Pietsch *et al.*, 2004 ▸; Meduňa *et al.*, 2009 ▸) with large angles of diffraction, or grazing incidence diffraction (GID) (Stepanov & Köhler, 1994 ▸) with small incidence angles but large diffraction angles, is used for strain investigation of nanomaterials in thin films. Each of these techniques has its advantages and disadvantages and is used for a specific purpose.

Measurement of RSMs is usually performed by varying the beam, sample or detector angles. This can be realized by a point detector or by a linear detector, where use of the latter can significantly speed up the measurement. With a 2D pixel detector the experiments can be done even more efficiently, since fewer mechanical movements of the goniometer are required. In most cases the RSMs are collected in two dimensions only, which is sufficient for the majority of experiments (Fewster, 1997 ▸). Mapping of reciprocal space in three dimensions is more demanding (Bauer *et al.*, 2015 ▸). For 3D mapping, 2D pixel detectors are even more indispensable since the collection of data would otherwise be extremely time consuming. But using a 2D detector requires a well collimated X-ray beam, which is usually only available at synchrotron sources. In a standard laboratory, we typically use a setup with line focus where the beam is collimated in only one direction within the scattering plane, so that the experiments are limited mostly to the coplanar geometry wherein the surface normal lies within the scattering plane.

Scattering experiments are symmetric in many cases, with the scattering vector oriented almost perpendicular to the sample surface. Rotating the sample around this direction then provides a collection of RSMs. The sample rotation around this axis also allows access to parts of reciprocal space which cannot be reached in a standard coplanar setup due to Ewald sphere limitations (Pietsch *et al.*, 2004 ▸; Yefanov, 2008 ▸), as is typical for GISAXS or GID geometries. Here we will show that the rotation of the sample around a certain fixed axis, when measured from various angular projections, can also be used in connection with the Radon transform even in the frame of reciprocal space.

The mathematical technique called the Radon transform (RT) originates from around 1917 ▸ when it was invented by Johann Radon (Radon, 1986 ▸). For many decades it has been successfully used in computerized tomography (CT) for medical and materials imaging (Buzug, 2008 ▸; Carmignato *et al.*, 2018 ▸). It is an integral transform applied for the mathematical 3D reconstruction of an object from a series of azimuthal projections (Herman *et al.*, 1991 ▸). While it is normally used for 3D X-ray imaging in real space, the Radon transform can also provide 3D RSMs under certain conditions in combination with the scattering techniques listed above.

In this work, we shall focus on the analysis of micro­structured semiconductor samples, mainly by mapping X-ray diffraction in 2D and 3D reciprocal space using a linear or a matrix pixel detector and a standard sealed X-ray tube with line focus. Analysing strain and surface morphology will be the main goal of the study. The standard structural analysis will be complemented by Radon transform processing in order to derive 3D RSMs under laboratory conditions. The samples used for this study consist of microcrystals of various materials grown epitaxially on large arrays of micropillars etched into Si substrates (Falub *et al.*, 2012 ▸). The epitaxial microcrystals consist of Ge, SiGe or GaAs (Falub *et al.*, 2013 ▸, 2014 ▸; Taboada *et al.*, 2016 ▸).

Closely spaced SiGe microcrystals with exceptionally high aspect ratios have been epitaxially grown by low-energy plasma-enhanced chemical vapour deposition (LEPECVD) (Rosenblad *et al.*, 1998 ▸) on Si pillars. Their growth enables us to overcome the defect problem associated with threading dislocations usually accompanying the misfit dis­locations generated during heteroepitaxial growth of lattice-mismatched layers, and provides a means of relieving thermal strain (Falub *et al.*, 2012 ▸; von Känel *et al.*, 2014 ▸; Isa *et al.*, 2016 ▸). Such materials follow the trend towards functional scaling of electronic devices according to the ‘more than Moore’ concept (Kent & Prasad, 2008 ▸).

Lattice strain and defects induced by growing lattice-mismatched materials at elevated temperatures can be studied by X-ray scattering techniques, in particular by X-ray diffraction RSMs (Rozbořil *et al.*, 2016 ▸; Meduňa, Falub *et al.*, 2018 ▸; Meduňa *et al.*, 2019 ▸). The mapping of lattice bending in three dimensions inside micro- or nanostructures is typically a task for laboratories equipped with synchrotron radiation sources where a collimated beam is available. A series of such experiments has been performed previously on the same or similar samples (Falub *et al.*, 2013 ▸; Meduňa *et al.*, 2014 ▸, 2016 ▸; Meduňa, Isa *et al.*, 2018 ▸). In the present work we show that 3D RSMs obtained with standard laboratory diffractometers and with reconstruction by means of the RT are comparable to the 3D RSMs obtained at a synchrotron source (Meduňa *et al.*, 2014 ▸). The only differences are that the laboratory X-ray tube provides a slightly lower data quality and requires a slightly longer measurement time.

## 2. Mapping reciprocal space

**Figure 1:**
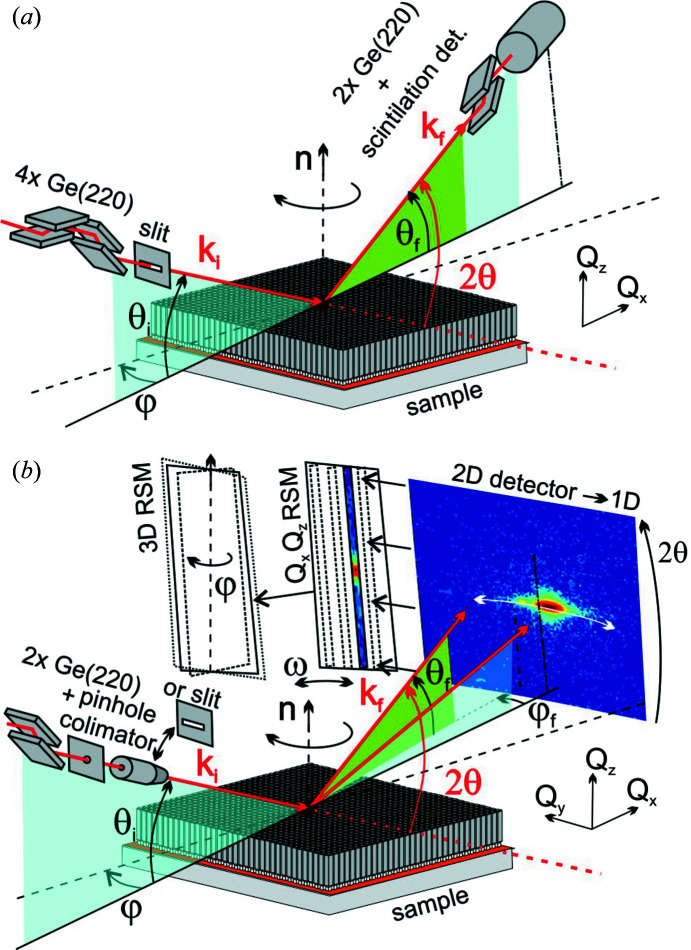
Sketches of the experimental schemes for obtaining (*a*) ultrahigh-resolution RSMs in two dimensions with an analyser and scintillation detector and (*b*) low-resolution RSMs in three dimensions using a 2D pixel detector. The turquoise plane is formed by the primary beam **k**_i_ and surface normal **n** (coplanar scattering plane) and the green one denotes the plane perpendicular to the sample surface involving the [generally non-coplanar (*b*)] output beam **k**_f_. The 2D (*Q*_*x*_, *Q*_*z*_) RSMs are built from 2θ scans/shots of the pixel detector used in 1D mode with rocking angle ω = (θ_i_ − θ_f_)/2. The 2D maps recorded under different azimuths φ can form a 3D RSM.

To analyse strain inside semiconductor microcrystals we use HRXRD mapped in reciprocal space primarily in two dimensions. The measurements were taken with a Rigaku diffractometer equipped with a Cu-based rotating anode (45 kV, 180 mA), a parabolic multi-layer mirror and an additional monochromator. For ultrahigh resolution, the parallel and monochromatic beam is defined by a four-bounce Ge(220) Bartels-type monochromator placed after the mirror, where the typical beam size defined by slits is 5 × 1 mm. The signal is collected by a scintillation counter with a channel-cut Ge(220) analyser placed in front of the detector in the ultrahigh-resolution setup shown in Fig. 1 ▸(*a*).

Other high-resolution measurements, where higher intensity is required, are realized with a slightly decreased resolution using only a two-bounce channel-cut Ge(220) monochromator. The 2 × Ge(220) channel-cut monochromator transmits the single Cu *K*α_1_ spectral line with a resolution Δλ/λ = 0.03% typical for a Cu anode (Melia *et al.*, 2019 ▸). The angular resolution of the incident beam is Δθ_i_ ≃ 0.01° for the 2 × Ge(220) channel-cut monochromator and Δθ_i_ ≃ 0.003° for the 4 × Ge(220) Bartels monochromator. These parameters of the incident beam are sufficient to resolve the Si(004) substrate peak, for instance. Further resolution limits come from the side of the detector along 2θ.

In order to detect more intensity extended towards the third dimension of reciprocal space, we use a slightly different experimental setup. The necessity of having sufficient intensity limits the new setup to the somewhat lower resolution provided by a two-bounce Ge(220) channel-cut monochromator. In this case we use a 2D pixel detector (Rigaku HyPix 3000) in order to detect signal diffracting out of the scattering plane (the turquoise plane spanned by the incident beam and surface normal in Fig. 1 ▸) as well, which offers the possibility to record 3D RSMs directly or using azimuthal rotation of the sample and further data processing, as shown in Fig. 1 ▸(*b*). If non-coplanar resolution is required, the Ge monochromator is also followed by a pinhole collimator of diameter 0.2 mm, in order to limit the divergence of the primary beam. If the 2D pixel detector is used in a 1D mode (along 2θ), linear slits can be used as well for higher intensity. Note that in Fig. 1 ▸(*b*) and in the following discussion the co­ordinate perpendicular to the scattering plane is labelled *Q*_*y*_.

As seen in Fig. 1 ▸, the monochromated and collimated primary beam **k**_i_ generally hits the sample surface with the microcrystal array at an angle θ_i_ and the diffracted beam **k**_f_ exits the sample surface at an angle θ_f_ under the diffraction angle 2θ = θ_i_ + θ_f_, generally deviating from the scattering plane by an angle φ_f_, if a non-coplanar setup is realized. Then, the scattering vector **Q** = **k**_f_ − **k**_i_ defines the reciprocal-space coordinates (*Q*_*x*_, *Q*_*y*_, *Q*_*z*_) of the intensity RSM. When the coplanar RSM (*Q*_*x*_, *Q*_*z*_) is measured, the sample is rocked around ω = (θ_i_ − θ_f_)/2, changing the incidence angle θ_i_ with 2θ kept constant, and the 2D pixel detector is used in linear (1D) mode whereby the intensity is integrated along the direction *Q*_*y*_ ≃ φ_f_. Here the linear slit after the monochromator can be used. The 3D RSM can be built from the 2D RSMs, when the whole sample is rotated by the azimuthal angle φ around the surface normal [or around the (*h*, *k*, *l*) vector]. The 3D RSM is then constructed from series of (*Q*_*x*_, *Q*_*z*_) RSMs recorded at different azimuths (see Section 4 below). If the (*Q*_*x*_, *Q*_*y*_, *Q*_*z*_) RSM is built directly from the 2D detector pixel matrix, where ω is varied and both the θ_f_ and the φ_f_ angles correspond to the position on the sensor, the pinhole collimator must be used and the intensity is strongly reduced.

For a collimated beam, the divergence perpendicular to the scattering plane Δφ ≃ 0.08° is due to the pinhole setup and Δθ_i_ ≃ 0.01° in the scattering plane due to the channel-cut monochromator [Fig. 1 ▸(*b*)]. Using the linear slit after the channel-cut monochromator, the horizontal divergence Δφ along *Q*_*y*_ is high and can be more than several degrees (Δφ ≃ 4°, Δ*Q*_*y*_ ≃ 0.3 Å^−1^). Since the HyPix detector pixel size is 0.1 × 0.1 mm, the resolution at the diffracted beam position is approximately Δθ_f_ = Δφ_f_ ≃ 0.02°. Using the ultrahigh-resolution setup with a Bartels monochromator and analyser crystal in front of the scintillation detector we obtain Δθ_i_ ≃ 0.003° and Δθ_f_ ≃ 0.01°, whereas the beam is strongly divergent along *Q*_*y*_, so the *Q*_*y*_ coordinate is not resolved at all.

The RSMs presented in this work will serve mainly for the strain analysis of epitaxial microcrystals on Si composed of different materials. We shall discuss results derived from 2D RSMs, and later from 3D RSMs, collected in a common laboratory setup. We first present a typical analysis of strain and relaxation of extremely high aspect ratio microcrystals, and then move to the shape of RSM peaks in 3D reciprocal space, on which the application of the RT technique in reciprocal space will be demonstrated.

## 3. High-aspect-ratio SiGe microcrystals

High-aspect-ratio SiGe microcrystals have already been studied elsewhere (Rovaris *et al.*, 2017 ▸; Montalenti *et al.*, 2018 ▸). The ones investigated here are special because of their height, which exceeds tens of micrometres. We measure RSMs for two types of sample with a nominal Ge content of 85%: SIGE40 microcrystals with a height of 40 µm and a width of 6 µm were grown onto 2 µm wide Si pillar bases surrounded by 4 µm wide trenches, and SIGE100 microcrystals with a height of 100 µm and a width of 7 µm were grown onto Si pillars 2.5 µm wide and 4.5 µm apart. These dimensions show that the microcrystals are nearly space filling, as a result of lateral expansion during growth, by which the gaps between neighbours shrink to just a few nanometres. The sample details are described in more detail by Meduňa *et al.* (2021 ▸). Surface diffusion at the elevated growth temperature of around 813 K and the tiny gaps and rough sidewalls cause the microcrystals to start coalescing, typically at a height of around 30 µm. The cracks formed during cooling because of the different thermal expansion coefficients of Si and Ge have been the subject of another paper wherein these cracks and the bending of the crystal lattice were visualized by rocking-curve imaging (Meduňa *et al.*, 2021 ▸).

**Figure 2:**
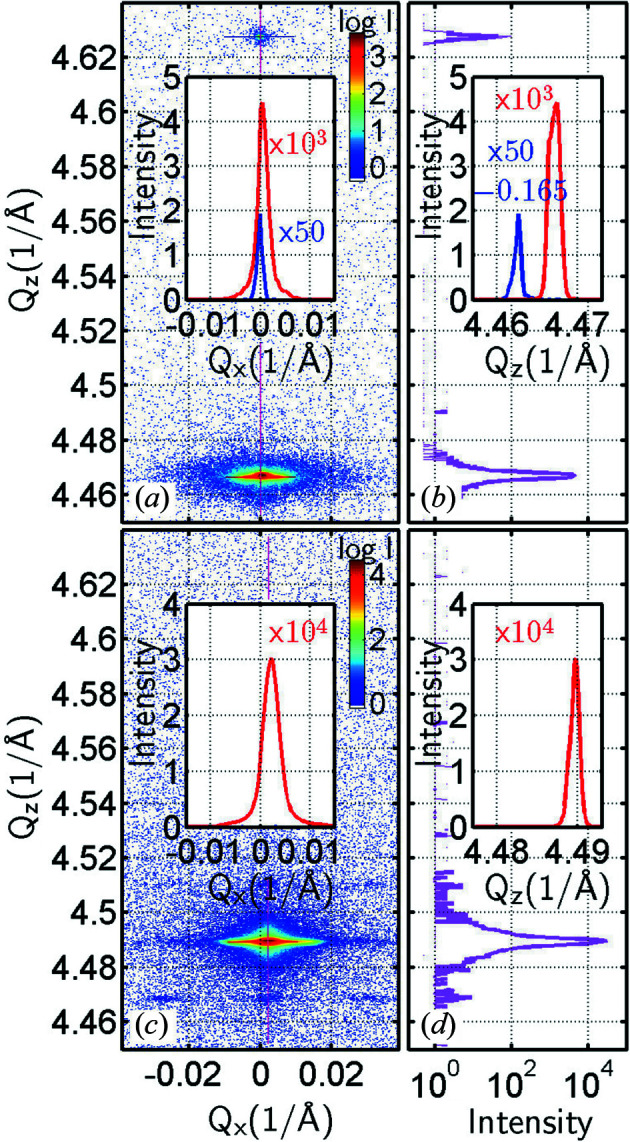
Symmetric (004) RSMs for (*a*), (*b*) sample SIGE40 and (*c*), (*d*) sample SIGE100, together with line cuts along the *Q*_*z*_ axis [panels (*b*) and (*d*) on a logarithmic scale and their insets on a linear scale] and along the *Q*_*x*_ axis [insets in panels (*a*) and (*c*) on a linear scale]. For sample SIGE40 a Ge peak (red) is shown together with the Si peak (blue). Note that in the inset in panel (*a*) the Si peak position has been shifted by Δ*Q*_*x*_ = −0.165 towards the Ge peak and its intensity magnified by a factor of 50 in order to make the Si and Ge peaks comparable. For sample SIGE100, the Si peak (blue) was not detected in the high-resolution setup due to high absorption in the 100 µm Ge microcrystals.

In order to determine precisely the Ge content inside the microcrystals, their average strain and their degree of relaxation, we have measured RSMs around the (004) and (115) Si and SiGe reciprocal-lattice points using the HRXRD setup of Fig. 1 ▸(*a*) in both perpendicular azimuths [110] and [ 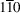 ]. An example of a symmetric (004) RSM is shown in Fig. 2 ▸ for both investigated samples SIGE40 and SIGE100. Intensity cuts through the Si and SiGe peaks along *Q*_*z*_ in the right-hand panels of Fig. 2 ▸ demonstrate the crystalline quality of the epitaxial SiGe since the SiGe peak is only slightly wider than the substrate peak. The shape and peak widths and their correspondence to misfit dislocations in the microcrystals are discussed further later in this section. As the SiGe layer composed of elongated tall microcrystals is very thick, the observed Si peak is very low in intensity due to the absorption of X-rays inside the SiGe. For sample SIGE100, the beam is completely absorbed in the 100 µm thick Ge region, making the reference Si peak invisible in this setup. Using only the two-bounce Ge monochromator, the Si peak was visible. With our experience from other measurements, we could therefore safely assume nominal Si peak positions for the strain analysis.

From the (004) RSM shown in Fig. 2 ▸ and the corresponding (115) RSM, we found that the Ge content in the microcrystals of sample SIGE40 is *x* = 0.877 ± 0.002. A similar analysis gave *x* = 0.764 ± 0.002 for sample SIGE100. The average in-plane strains found in the microcrystals were ɛ_||_ = (−3.7 ± 1.2) × 10^−4^ for sample SIGE40 and ɛ_||_ = (−2.1 ± 1.2) × 10^−4^ for sample SIGE100. The average normal strains were ɛ_⊥_ = (2.8 ± 0.9) × 10^−4^ for sample SIGE40 and ɛ_⊥_ = (5.0 ± 2.7) × 10^−4^ for sample SIGE100. The strains are defined as ɛ_||_ = (*a*_SiGe_ − *a*_||_)/*a*_SiGe_ and ɛ_⊥_ = (*a*_SiGe_ − *a*_⊥_)/*a*_SiGe_, where *a*_||_ and *a*_⊥_ are the horizontal and vertical lattice parameters, respectively, and *a*_SiGe_ is the lattice parameter of the unstrained bulk material The degrees of relaxation *R* = (*a*_||_ − *a*_Si_)/(*a*_SiGe_ − *a*_Si_) of the SiGe layers were *R* = 1.010 ± 0.004 for sample SIGE40 and *R* = 1.007 ± 0.004 for sample SIGE100. The SiGe microcrystals are hence slightly over relaxed because of thermal strain induced by cooling from the growth temperature to room temperature. These results for the strain are consistent with our previous studies of shorter Ge and SiGe microcrystals (Falub *et al.*, 2012 ▸, 2013 ▸; Rozbořil *et al.*, 2016 ▸; Meduňa, Falub *et al.*, 2018 ▸).

Intensity cuts across the diffraction peaks in Fig. 2 ▸ allow us to compare the SiGe and Si peak widths as well, which being very close to each other prove the good crystallinity. We can compare the FWHM values using the HRXRD RSMs, where we obtain for the SiGe peak Δ*Q*_*x*_ = (7.48 ± 0.09) × 10^−4^ Å^−1^ and Δ*Q*_*z*_ = (6.81 ± 0.10) × 10^−4^ Å^−1^ for the SIGE40 sample, and Δ*Q*_*x*_ = (14.49 ± 0.40) × 10^−4^ Å^−1^ and Δ*Q*_*z*_ = (5.66 ± 0.09) × 10^−4^ Å^−1^ for the SIGE100 sample. The peak aspect ratios are Δ*Q*_*z*_/Δ*Q*_*x*_ = 0.91 ± 0.02 for SIGE40 and Δ*Q*_*z*_/Δ*Q*_*x*_ = 0.39 ± 0.04 for SIGE100. The values for the Si peak are Δ*Q*_*x*_ = (5.45 ± 0.05) × 10^−4^ Å^−1^ and Δ*Q*_*z*_ = (3.96 ± 0.15) × 10^−4^ Å^−1^ so that Δ*Q*_*z*_/Δ*Q*_*x*_ = 0.73 ± 0.03. For the majority of 60° misfit dislocations (Kaganer *et al.*, 1997 ▸), as mostly expected in these microcrystals (Falub *et al.*, 2012 ▸, 2014 ▸), the aspect ratio of the FWHM is Δ*Q*_*z*_/Δ*Q*_*x*_ = 0.3. If the ratio is smaller, we assign the peak broadening to further misorientation of the lattice planes along the *Q*_*x*_ direction caused by mosaicity or by lattice bending originating from thermal strain (Rozbořil *et al.*, 2016 ▸; Meduňa, Falub *et al.*, 2018 ▸). For the present crystals, by contrast, the aspect ratio is >0.3, which can most probably be attributed to a more complicated defect distribution. These crystals start to merge during growth at a height of around 30–40 µm and then split into five rather irregular crystals growing independently. After cooling from the growth temperature the arrays of microcrystals form cracks, as seen by Meduňa *et al.* (2021 ▸).

**Figure 3:**
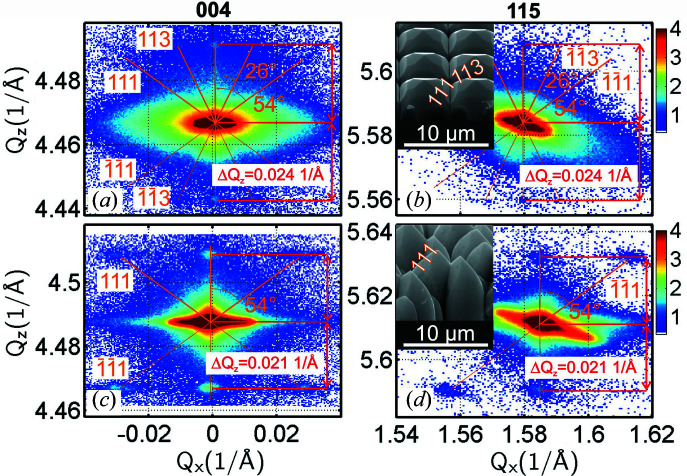
(*a*), (*c*) Symmetric (004) and (*b*), (*d*) asymmetric (115) RSMs of (*a*), (*b*) sample SIGE40 and (*c*), (*d*) sample SIGE100, showing the side maxima along the 〈111〉 directions for both samples and along the 〈113〉 directions for SIGE40 coming from crystal facets on top of the microcrystals, illustrated in the SEM images in the insets of panels (*b*) and (*d*). The lateral maxima positions are determined by streak deviation and by Δ*Q*_*z*_ distance from the main central peak.

Additionally, further lateral satellite maxima around the symmetric (004) diffraction point are observed for sample SIGE100 in Fig. 2 ▸ measured with the high-resolution setup. In order to resolve these satellites even better, we recorded the 2D RSMs around the symmetric (004) and asymmetric (115) SiGe diffraction points for both the SIGE40 and SIGE100 samples using the lower reciprocal-space resolution and higher-intensity setup as shown in Fig. 1 ▸. These detailed RSMs are shown in Fig. 3 ▸, together with scanning electron microscopy (SEM) perspective views of the SiGe microcrystals, and we observe that such lateral satellites are present in both samples around the symmetric and asymmetric SiGe diffraction points.

The satellites are present at two fixed *Q*_*z*_ positions vertically symmetric around the main SiGe peak in the centre. At both of these *Q*_*z*_ positions there are three maxima, a central one at the same *Q*_*x*_ as the main peak, and two symmetrically placed lateral ones present when measured along the [110] sample edges at azimuth φ = 0°. The lateral distance Δ*Q*_*x*_ of the satellites differs significantly for SIGE40 and SIGE100, whereas the vertical distance Δ*Q*_*z*_ from the main peak is very similar for both samples, Δ*Q*_*z*_ = 0.024 Å^−1^ for SIGE40 and Δ*Q*_*z*_ = 0.021 Å^−1^ for SIGE100. The satellite positions Δ*Q*_*x*_, Δ*Q*_*z*_ measured relative to the main diffraction peak are the same for (004) as for (115) RSMs, but the intensity distribution of individual satellites is slightly different for (115) compared with (004) due to lateral strain sensitivity.

A detailed inspection of the RSMs reveals that, next to the above-mentioned satellites, a slightly enhanced intensity in the form of streaks pointing from the central SiGe peak towards the satellites is present. These streaks correspond perfectly to the truncation rods of the {111} and {113} (SIGE40 only) facets that are also observed in the SEM micrographs in the insets of Figs. 3 ▸(*b*) and 3 ▸(*d*). In the smaller microcrystals of SIGE40 the {113} facets dominate [see the SEM image in Fig. 3 ▸(*b*)]; thus the streak at 26° deviation from [001], corresponding to the [113] direction, is more significant. Some streaks at 54° deviation from [001], corresponding to the 〈111〉 directions, are also slightly visible since the {111} facets are present as well in SIGE40. By contrast, on the tall SIGE100 microcrystals the {113} facets are practically missing in the SEM image in Fig. 3 ▸(*d*), and thus only the streaks at 54° deviation from [001], corresponding to the 〈111〉 directions, are visible. In fact, the satellites are formed as intersections of the streaks and the ±Δ*Q*_*z*_ planes, so the satellites are more representative than the streaks due to their better visibility. The satellites are laterally much closer in SIGE40 and more distant in SIGE100, since their lateral position is driven by streak inclination and thus determined by microcrystal surface facet orientations as well. What governs the vertical positions ±Δ*Q*_*z*_ of the planes of higher intensity is unfortunately not well known. One possibility is that the intensity there originates from some vertical period­icity of the strain due to the defects having a spatial period of *L*_*z*_ = 26 nm in SIGE40 and *L*_*z*_ = 30 nm in SIGE100. Another possibility is that it may arise from some interlayer of different Ge composition inside the microcrystals. This is, however, unlikely in view of the symmetric position ±Δ*Q*_*z*_ of these planes around the main peak.

Because the lateral separation between the satellites is determined as much by the vertical positions ±Δ*Q*_*z*_ as by the orientation of truncation rods from fourfold symmetric facets on the microcrystal surfaces, the idea arises that each satellite in an RSM must belong to an individual facet. Some of them can even be assigned to more than one facet, since the satellites can overlap each other as they are projected along the *Q*_*y*_ axis to the same positions in the *Q*_*x*_ *Q*_*z*_ RSM, as also previously published for other microcrystals (Meduňa *et al.*, 2016 ▸). This is the reason why we observe only two lateral and one central satellite instead of four, since the central one is a superposition of two overlapping maxima when measured along [110] at azimuth φ = 0°. This can be clearly observed when the RSMs are recorded at azimuths slightly different from 〈110〉, because the middle satellites around *Q*_*x*_ = 0 will then split into two and four satellites are detected, as will be shown in Section 4.

An azimuthal rotation of RSMs around the surface normal can give rise to a Radon transform performed on a series of different projections in the investigated reciprocal space. If we measure RSMs, for instance, around the (004) diffraction point at various azimuths φ of the scattering plane using linear slits along the *Q*_*y*_ direction, the intensity is always summed along the *Q*_*y*_ axis and recorded in the *Q*_*x*_ *Q*_*z*_ plane as a projection. The reconstruction of the 3D reciprocal space from these 2D projections described in the next section for SiGe microcrystal samples is in fact similar to the standard Radon transform carried out in real space.

## 4. RSMs and the Radon transform

Typical mapping of scattered intensity in reciprocal space using standard laboratory equipment with a line-shaped (*i.e.* formed from linear focus using linear slits) collimated monochromatic beam and a point or linear detector involves measurement of RSMs only in two dimensions, particularly in *Q*_*x*_ *Q*_*z*_ coordinates. Since the intensity is usually blurred along the *Q*_*y*_ direction perpendicular to the scattering plane, due to low beam collimation along the larger side of the beam cross section, the whole intensity signal 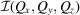 at a certain RSM point (*Q*_*x*_, *Q*_*z*_) is integrated over *Q*_*y*_, and the resulting *Q*_*x*_ *Q*_*z*_ RSM can be understood as a projection of the 3D reciprocal space (*Q*_*x*_, *Q*_*y*_, *Q*_*z*_) along the *Q*_*y*_ direction (Meduňa *et al.*, 2014 ▸, 2019 ▸).

One of the possibilities for recording the complete RSM in three dimensions is to collimate the primary beam not only within the scattering plane (*Q*_*x*_, *Q*_*z*_) but also perpendicular to it, which requires either a synchrotron beam, or pinhole or special optics, such as polycapillary or Montel optics (Hertlein *et al.*, 2005 ▸; He, 2018 ▸). In any case, this leads to a diminished intensity contributing to the scattering process. The second approach to obtain information about the scattered intensity in 3D reciprocal space is to record 2D slices through the probed reciprocal region at various azimuthal positions, and to build the 3D intensity distribution by a mathematical transformation. Such a mathematical method, called the Radon transform, is usually applied to real-space coordinates and used in CT for medical or materials imaging in real space (Suetens, 2009 ▸).

**Figure 4:**
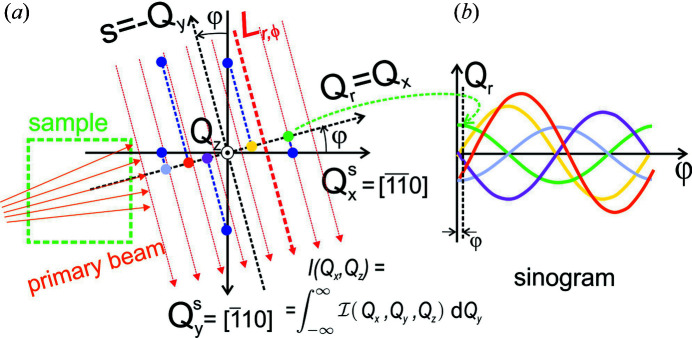
(*a*) A geometric scheme of reciprocal-space mapping under various azimuths using a highly divergent X-ray beam (orange arrows) incident on the sample (green square) and analogous to the Radon transform used in standard CT techniques. (*Q*_*r*_,*s*) represent the reciprocal coordinate system bound with the diffractometer and 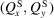 is fixed with the sample. Each of the blue points is projected onto a coloured point on the *Q*_*r*_ axis along the *L*_*r*,φ_ line determined by *Q*_*r*_ position and direction φ. When the sample is rotated, the coloured points move along the *Q*_*r*_ axis as a sine function, building an intensity distribution in a sinogram map. (*b*) Examples of sinograms *I*(*Q*_*r*_,φ) corresponding to the coloured points projected along the *L*_*r*,φ_ line in panel (*a*).

In this work, we will also apply the Radon transform, but realize it in reciprocal space. Instead of using the X-ray linear attenuation factor μ(*x*, *y*) as a transform function in real space we will use the distribution of the scattered intensity 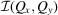 recorded for different reciprocal slice positions *Q*_*z*_. A scheme of the projection geometry showing the analogy between reciprocal-space mapping and CT in real-space imaging is shown in Fig. 4 ▸. Recall that in standard real-space CT the projection is realized along the parallel primary beam (*s* direction) (Suetens, 2009 ▸). Here, in mapping the RSM the projection is realized along *Q*_*y*_, *i.e.* perpendicular to the quasi-divergent primary beam.

In CT using the method of RT (Suetens, 2009 ▸) we will introduce coordinates similar to the standard Radon transform, *r* ≡ *Q*_*r*_ = *Q*_*x*_ and *s* = −*Q*_*y*_. We also introduce the coordinate system bound to the probed sample 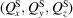 with 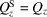 since the sample is rotated around the surface normal. This gives coordinate transformation formulas 

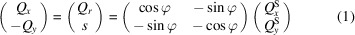

and the inverse 

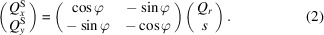

For a fixed azimuth angle φ at fixed *Q*_*z*_, and for a large beam divergence or wide horizontal (along *Q*_*y*_) acceptance of the detector, the measured intensity profile as a function of *Q*_*x*_ ≡ *Q*_*r*_ is given by 

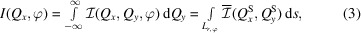

where 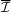 is the intensity in the coordinate system of the sample and *L*_*r*,φ_ is a line at a distance *Q*_*r*_ from the origin subtending an angle φ with the 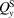 axis. The divergence must cause the beam to span across the detector so that the range Δ*Q*_*y*_ is larger than the whole range of the recorded intensity. As mentioned in Section 2, the horizontal beam divergence with a linear slit was Δφ ≃ 4°, corresponding to Δ*Q*_*y*_ ≃ 0.3 Å^−1^, which is much larger than any RSM range shown in this paper. On the other hand, equation (3) is still valid when a small divergence using a pinhole is used, but a large detector acceptance must integrate the scattered intensity over a sufficiently large range Δφ_f_ covering the whole intensity structure along *Q*_*y*_ in this case. Thus the integral over *Q*_*y*_ can be realized by varying either of Δφ and Δφ_f_ or both.

We note that equation (3) holds only for the kinematic approximation, far from regions where refraction and dynamic effects are usually included (Pietsch *et al.*, 2004 ▸). Equation (3) can be rewritten in the form of a Radon transform performed in reciprocal space, 

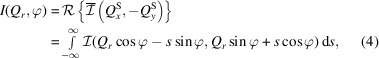

where *I*(*Q*_*r*_, φ) is the intensity projected onto the given scattering plane. Because the measurements at opposite azimuths (φ → φ + π) should give the same results in the kinematic approximation, it is usually sufficient to measure *I*(*Q*_*r*_, φ) for φ ranging from 0 to π. If we group the projections for all measured φ, we build a 2D map in the form of a sinogram as in Fig. 4 ▸(*b*). If the intensity has just the form of single dots at certain positions 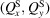 [blue circles in Fig. 4 ▸(*a*)], the projections onto the *Q*_*r*_ axis (coloured circles) will move in a sinusoidal way along this axis rotation angle φ, from which the sinogram derives its name. As an example, the coloured sine curves in Fig. 4 ▸(*b*) correspond to the trajectories along *Q*_*r*_ of the points of the same colour in Fig. 4 ▸(*a*). Their positions in Fig. 4 ▸(*a*) correspond to the φ value shown by the vertical black dashed line in Fig. 4 ▸(*b*).

In order to realize the inverse RT numerically on real measured data, the filtered back-projection process is usually applied, which is a combination of 1D and 2D fast Fourier transforms (FFTs) combined with resampling of the data from polar to Cartesian coordinates and with a frequency filter, as in a standard tomographic process (Suetens, 2009 ▸). The disadvantage of the direct Fourier reconstruction is higher uncertainty in the image due to possible artefacts formed, although this can be somewhat reduced by including a ramp filter in the FFT process which helps to compensate in­adequate spatial frequencies. After the application of a Ram-Lak filter we obtain slightly sharper images, but this can be inappropriate for large intensities as we will see later. For numerical processing we use the MATLAB programming environment with a built-in Radon transform routine.

**Figure 5:**
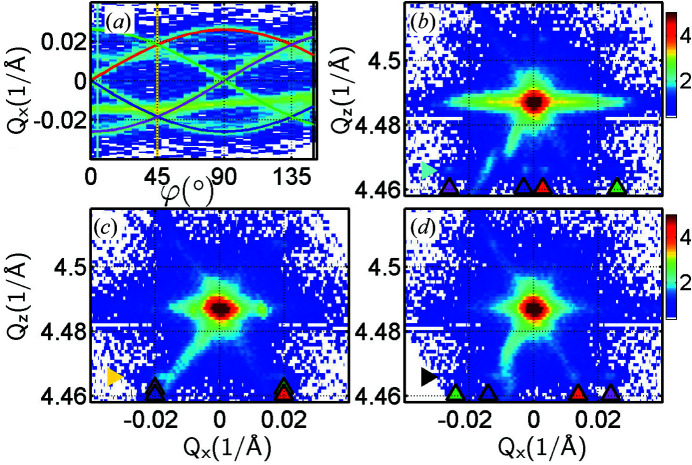
Symmetric (004) RSMs of the sample SIGE100 measured at different azimuths φ = 5, 45 and 150°, together with a sinogram at *Q*_*z*_ = 4.467 Å^−1^. (*a*) The sinogram shows four sine curves originating from four satellites. (*b*)–(*d*) The RSMs were reconstructed from series of 2θ scans recorded by rocking the sample using the signal from the 2D detector integrated along *Q*_*y*_. The triangles pointing to the satellites at *Q*_*z*_ = 4.467 Å^−1^ correspond to the sine curves of the same colour in the sinogram in panel (*a*).

A real example of a measured intensity sinogram in reciprocal space at a fixed *Q*_*z*_ position built from RSMs of sample SIGE100 rotated by φ around the surface normal is shown in Fig. 5 ▸(*a*) for *Q*_*z*_ = 4.467 Å^−1^, selected to show the lower satellite maxima. Figs. 5 ▸(*b*)–5 ▸(*d*) further demonstrate examples of typical (*Q*_*x*_, *Q*_*z*_) RSMs recorded at various selected azimuths φ, where the individual satellites shown by coloured triangles move along the chosen *Q*_*z*_ level as the projection plane rotates. The satellites then create the sine curves, marked by the same colour as the corresponding triangle, in the sinogram map in Fig. 5 ▸(*a*). A sinogram recorded for a sufficient density of azimuthal positions in the interval (0, π) can include enough information to build the intensity in the whole (*Q*_*x*_, *Q*_*y*_) plane. Sinograms built for all *Q*_*z*_ positions can then be used to reconstruct the whole 3D RSM by means of a filtered back-projection within the inverse RT.

**Figure 6:**
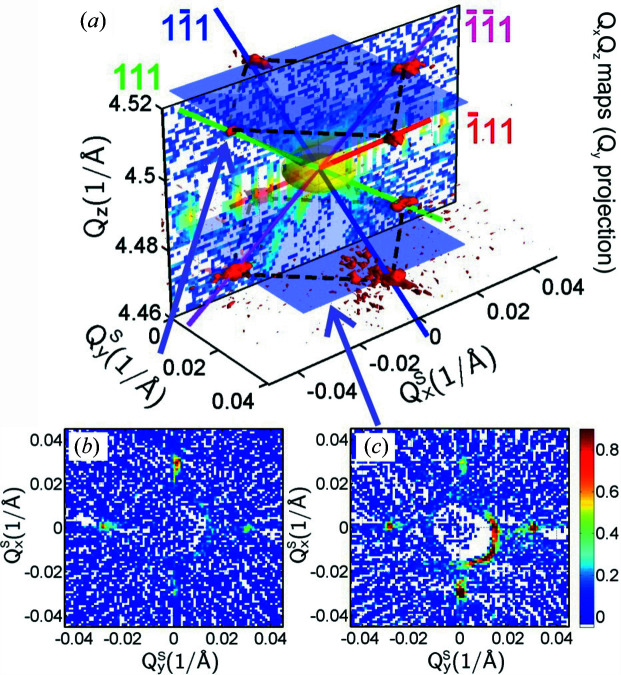
(*a*) A perspective view of the 3D symmetric (004) RSM of sample SIGE100 reconstructed using the Radon transform from *Q*_*x*_ *Q*_*z*_ maps recorded under different azimuths with the HyPix linear detector. Directions perpendicular to the 〈111〉 facet planes are enhanced by coloured lines. (*b*), (*c*) Plots of plane slices 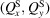 through the 3D map at positions (*b*) *Q*_*z*_ = 4.507 Å^−1^ and (*c*) *Q*_*z*_ = 4.467 Å^−1^ through the satellite maxima.

In Fig. 6 ▸(*a*) we plot such a 3D RSM reconstructed by means of Radon transforms using equation (4) applied to sinograms at different *Q*_*z*_ positions [see the example for fixed *Q*_*z*_ in Fig. 5 ▸(*a*)] for sample SIGE100. The 3D RSM was built from 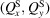 slices at *Q*_*z*_ positions from 4.46 to 4.52 Å^−1^. Examples of these slices for the lower and upper satellite positions are demonstrated in Figs. 6 ▸(*b*) and 6 ▸(*c*). Even though the 2D HyPix detector was used, the scanning was done in 1D detector mode, so that data collection was equivalent to that of a linear detector. We could also use 0D detector scanning along 2θ while collecting data but with much longer measurement times.

Unfortunately, some unwanted features originating from the characteristic resolution function of the setup, such as monochromator or detector streaks, can adversely affect the 3D RSM reconstruction. In order to accelerate the RSM measurements significantly at 36 azimuth positions and to enhance the intensity, the low-resolution setup was used for this experiment and thus both streaks are present in the RSMs of Figs. 5 ▸(*b*)–5 ▸(*d*). We also note here that the number of azimuthal positions (here 36, *e.g.* given by an azimuthal step of 5°) determines the resolution in reciprocal space within the reconstructed *Q*_*x*_ *Q*_*y*_ plane, depending primarily on the distance from (0, 0, *Q*_*z*_). So the larger the *Q* size area of the reconstructed objects in reciprocal space, the more azimuthal slices (smaller azimuthal steps) are necessary. The situation is similar to standard tomography in real space where the necessary azimuthal resolution is related to the size of the reconstructed area, but here in reciprocal-space units.

Using a Bartels monochromator, and in particular an analyser crystal, would eliminate the characteristic streaks in the RSMs, but this is realizable only with a point detector which strongly slows down the measurements. On the other hand, the streaks practically do not depend on the sample orientation and so are imprinted onto the sinograms as almost horizontal or slightly inclined lines (as the diffraction peak position is always recalibrated to the nominal position). This effect appears in the Radon transform as a central circle around the origin 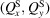 = (0, 0). As the streaks are individually inclined in (*Q*_*x*_, *Q*_*z*_) RSMs, the circle radius changes for different *Q*_*z*_, as can be seen in Figs. 6 ▸(*b*) and 6 ▸(*c*).

The reconstructed 3D RSM in Fig. 6 ▸(*a*) shows that the lower and upper satellites have fourfold symmetry, which exactly matches with the top {111} facets of the microcrystals. The satellites lie on the truncation rods perpendicular to the facets. The 〈111〉 directions [coloured lines in Fig. 6 ▸(*a*)] are inclined by 54° from [001], as discussed in Section 3. The fourfold symmetry is also clearly visible in the map slices of Figs. 6 ▸(*b*) and 6 ▸(*c*), since the four satellites are pronounced and sharp and the ring from the detector or monochromator streaks does not disturb the images significantly.

A problematic situation occurs when the intensity in the processed *Q*_*z*_ slice is extremely large in its dynamic range, for instance when the main diffraction peak is present at the considered *Q*_*z*_ level. When the central diffraction peak dominates in the *Q*_*x*_ slice and in the corresponding sinogram, the Radon transform is not able to retrieve the 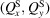 slice properly and the technique is very insensitive to side maxima present at the same *Q*_*z*_. The intensity around the intense peak is modulated by high noise, mostly due the numerical processing and filtering. On the other hand, without the application, for instance, of the Ram-Lak filter, the reconstructed 3D intensity is even more distorted. Thus the central (004) diffraction peak was always omitted in these RT-processed 3D RSMs, including the strong diffuse scattering around the (004) peak stemming from the dislocation field inside the SiGe microcrystals [yellow ellipsoid in Fig. 6 ▸(*a*)] and the middle part around **Q** = (0, 0, 4.487) Å^−1^. Upon removing the most intensive part around the main (004) peak of Fig. 6 ▸(*a*), together with the weakest part including noise, the fourfold symmetric satellites due to the truncation rods originating from the crystal facets became much more visible.

**Figure 7:**
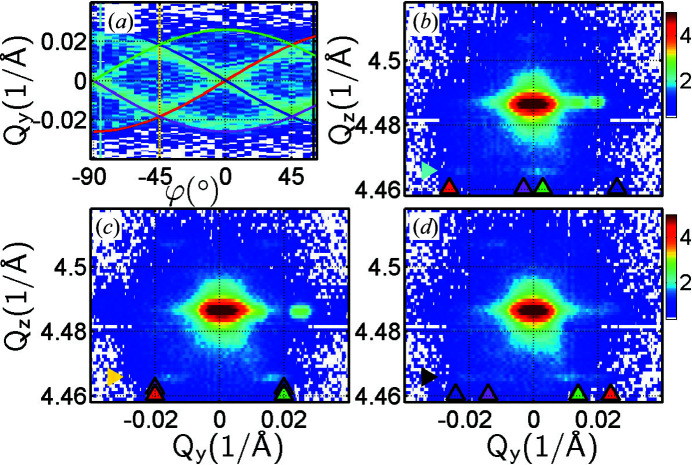
Symmetric (004) RSMs of sample SIGE100 measured at azimuths φ = −85, −45 and 60° together with a sinogram at *Q*_*z*_ = 4.467 Å^−1^. (*a*) The sinogram shows four sine curves originating from four satellites. (*b*)–(*d*) The RSMs were reconstructed from 3D RSMs built using shots of the HyPix 2D detector as the sample (ω) was rocked. The 2D *Q*_*y*_ *Q*_*z*_ RSMs were obtained as the signal was integrated along *Q*_*x*_ for individual pixels. The triangles pointing to the satellites at *Q*_*z*_ = 4.467 Å^−1^ correspond to the sine curves of the same colour in the sinogram in panel (*a*).

The 3D RSMs can be recorded directly using the 2D pixel-array detector, which also collects intensity from outside the scattering plane. The inconvenience is that the beam collimation must by very high in all directions so that data need to be collected for a longer time in order to preserve reasonable counting statistics. To test once more the application of the RT in reciprocal space, we collected a series of RSMs at different azimuths with the collimated beam. In this case the projection was realized numerically along the *Q*_*x*_ direction parallel to the beam. This is similar to the previous situation where the projection was done automatically along *Q*_*y*_ due to the beam divergence. The disadvantage here is that the resolution along *Q*_*y*_ is determined by the pinhole and the pixel size, and the better resolution along *Q*_*x*_ determined by the Ge monochromator is nearly lost. On the other hand, the advantage of this approach is that no detector or monochromator streaks are present in the *Q*_*y*_ *Q*_*z*_ plane. Since the projection path in this case is perpendicular to *Q*_*y*_, which was the projection direction in the previous cases of Figs. 5 ▸ and 6 ▸, we shift the azimuthal position appropriately in order to compare the two approaches. A new sinogram with φ shifted by −90°, together with selected 2D (*Q*_*y*_, *Q*_*z*_) RSMs, is shown in Fig. 7 ▸.

We note that the RSMs in Figs. 7 ▸(*b*)–7 ▸(*d*) do not include any detector streaks since these maps are built from 3D RSMs using shots of the HyPix 2D detector as the sample (ω) is rocked. The images from the HyPix are properly summed over the *Q*_*x*_ direction as the 2D *Q*_*y*_ *Q*_*z*_ RSMs are built. It is also evident that the satellite maxima from the {111} facets are elongated along the horizontal axis due to the lower collimation along *Q*_*y*_ because of the pinhole size. The vertical size is limited by the Ge monochromator before the pinhole. The intensity maps in Figs. 5 ▸ and 7 ▸ are otherwise very similar.

**Figure 8:**
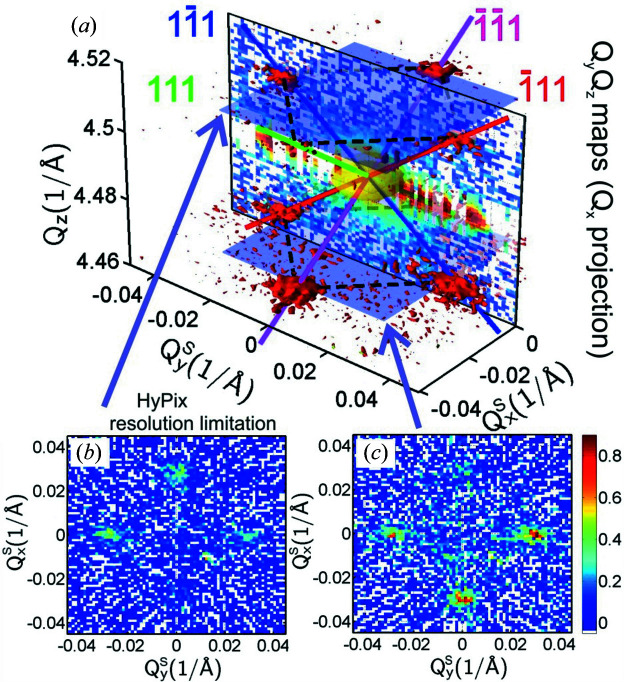
(*a*) A perspective view of the 3D symmetric (004) RSM of sample SIGE100 reconstructed using the Radon transform from *Q*_*y*_ *Q*_*z*_ maps recorded under different azimuths with the HyPix in a 2D mode, with the signal integrated along *Q*_*x*_ for individual pixels. Directions perpendicular to the 〈111〉 facet planes are enhanced by coloured lines. (*b*), (*c*) Plots of plane slices (*Q*_*x*_ *Q*_*y*_) through the 3D map at positions (*b*) *Q*_*z*_ = 4.507 Å^−1^ and (*c*) *Q*_*z*_ = 4.467 Å^−1^ through the satellite maxima.

In Fig. 8 ▸(*a*) we plot the 3D RSM reconstructed from 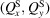 slices obtained by means of the RT using projections parallel to *Q*_*x*_ [see the *I*(*Q*_*y*_, φ) sinogram example in Fig. 7 ▸(*a*)]. As expected, the 3D isolevel RSM in Fig. 8 ▸(*a*) is practically identical to the one in Fig. 6 ▸(*a*), except that the 〈111〉 facet satellites are slightly broader compared with those in Fig. 6 ▸(*a*), due to the lower resolution of the pinhole along *Q*_*y*_. The same is also observed in the 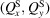 slices at *Q*_*z*_ for the lower and upper satellite positions demonstrated in Figs. 8 ▸(*b*) and 8 ▸(*c*). In these panels we also see that the circular artefact in the middle, originating from detector streaks, is missing. The orientation of both 3D plots in Figs. 6 ▸(*a*) and 8 ▸(*a*) is the same with respect to the sample so that they can be compared. Only the main 2D projection intensity maps 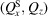 or 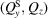 , respectively, differ according to the projection path for the RT which was *Q*_*y*_ or *Q*_*x*_, respectively.

These results of RSMs with simple fourfold symmetric satellites show that the RT is applicable for the reconstruction of 3D RSMs around symmetric diffraction points. If the dynamic range of the intensity inside the probed reciprocal area is rather low, the 3D intensity distribution is well reconstructed. If very high intensity peaks are present in the processed area, the technique has some weaknesses and the most intensive areas should be eliminated. If only a 1D detector is available and a linear slit is used, this procedure can save time because of the increased intensity during the collection of 3D RSMs. In the next section we will demonstrate the application of the RT to other more complex samples inspected on a standard diffractometer, and we will compare the results with synchrotron measurements recorded on samples with very similar structures.

## 5. SiGe multiple-quantum-well microcrystals

**Figure 9:**
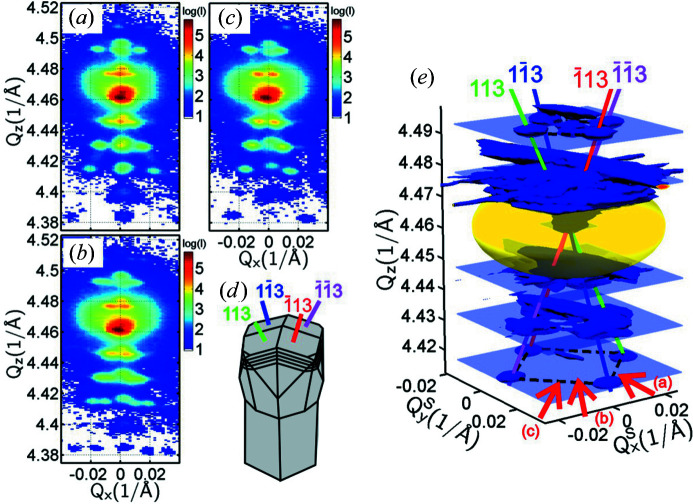
Symmetric (004) RSMs of the MQWs on SiGe microcrystals recorded at different azimuthal directions φ of the incidence plane for (*a*) φ = 0°, (*b*) φ = 30° and (*c*) φ = 44°. (*d*) The SiGe microcrystals with the SiGe/Si MQW structure are terminated by (113) facets which are responsible for the truncation rods along the [113] directions. (*e*) A perspective view of the 3D symmetric (004) RSM reconstructed using the Radon transform from *Q*_*x*_ *Q*_*z*_ maps recorded under different azimuths. It demonstrates the directions of truncation rods perpendicular to the crystal facets. The red arrows indicate the projection views of the maps in panels (*a*), (*b*) and (*c*).

The application of the RT to reciprocal space can also be realized on samples with more complex structures. As a further example we select arrays of epitaxial SiGe microcrystals containing Ge/SiGe multiple quantum wells (MQWs) near the top facets. The SiGe microcrystals were again grown by LEPECVD at a temperature of around 823 K, similarly to the SIGE40 and SIGE100 samples of Section 3, but only up to a height of around 8 µm. The microcrystals are terminated by {113} facets as shown in Fig. 9 ▸(*d*). Their lateral size is around 7 µm, resulting from lateral and vertical expansion during growth on 2 × 2 µm Si pillars separated by 5 µm gaps. The MQW structure was designed for particular luminescent properties (Pezzoli *et al.*, 2014 ▸). The same sample was previously studied by a scanning nanodiffraction technique at the ESRF. There we recorded 3D RSMs obtained from individual microcrystals directly by a nanobeam (Meduňa *et al.*, 2014 ▸). Here we apply the RT in reciprocal space in order to get a similar 3D RSM averaged over the whole array of microcrystals from a typical laboratory setup.

The sample with an array of MQW microcrystals was examined with the Rigaku diffractometer using the same setup as for the SIGE40 and SIGE100 samples. Series of 2D RSMs around the (004) diffraction point were collected under different azimuths with a step of Δφ = 2°. The azimuthal resolution in the (*Q*_*x*_, *Q*_*y*_) plane was chosen to be better than in Section 4 since the 3D RSM of the SiGe MQW sample includes many more features and structures in the intensity map than those for the SIGE40 and SIGE100 samples. A higher number of azimuthal positions (90) were chosen in this case despite the (*Q*_*x*_, *Q*_*y*_) area in the RSM being about two times smaller for the SiGe MQW sample than for SIGE40/100, but we generally preferred more detail overall. The 2D pixel detector was used in a linear mode since linear slits were applied in the primary beam. Examples of RSMs collected at azimuthal directions φ = 0, 30 and 44° of the scattering plane are shown in Figs. 9 ▸(*a*)–9 ▸(*c*). They show various MQW satellite peaks aligned along the inclined directions, giving information about the layer thicknesses of the periodic Ge/SiGe structure (Isa *et al.*, 2015 ▸) and a period of around 40 nm. Again, these directions correspond to the truncation rods perpendicular to the {113} microcrystal facets.

If only one RSM at φ = 0° is obtained, such as that in Fig. 9 ▸(*a*), one can get the impression that only three truncation rods are present and an additional dominant surface plane would be (001) parallel to the substrate surface. Therefore RSMs at additional azimuths are necessary. As the sample is rotated around the (004) *Q*_*z*_ axis, additional truncation rods will appear. If we select any azimuth φ ≠ 0, 45°, generally four truncation rods will be visible as in Fig. 9 ▸(*b*). In contrast, for azimuths very close to 45°, such as in the RSM measured at φ = 44° in Fig. 9 ▸(*c*), we apparently detect only two truncation rods.

This peculiar observation can be explained by the distribution of the truncation rods and MQW satellites in 3D reciprocal space and their projection onto the scattering plane, and this can be resolved only by azimuthal sample rotation. The measurement of RSMs for different azimuths and the reconstruction of the whole intensity distribution in three dimensions using the inverse RT can then provide us with an overall view of the scattered intensity. We have applied the inverse RT to about 90 RSMs, recorded for azimuths within the interval φ = 0–180°, and we have reconstructed the 3D RSM of the whole structure averaged over the array of MQW microcrystals. The result can be seen in Fig. 9 ▸(*e*), where the spatial alignment of the MQW satellite peaks along the four (113) truncation rods (red, green, blue and magenta lines) is clearly evident.

As seen in the 2D RSMs of Figs. 9 ▸(*a*)–9 ▸(*c*), representing the three azimuths indicated by red arrows in Fig. 9 ▸(*e*), strong diffuse scattering around the main GeSi peak originating from defects such as misfit dislocations is present with high intensity. This strong signal suppresses many details around the highest peak when realizing a 3D surface plot in order to give an overview of MQW satellites. Moreover, this high intensity around the peak is responsible for the difficulties encountered when the intensity is reconstructed by means of sinograms and using the inverse RT. As mentioned in Section 4, the application of filters will usually amplify the experimental noise, which, together with the dynamic intensity range, may make it impossible to obtain sinograms at certain *Q*_*z*_ positions. In order to eliminate the influence of high intensity on the 3D RSM reconstruction, the central part of the RSM around the main diffraction peak was thus eliminated [see the yellow ellipsoid in the central part of the RSM in Fig. 9 ▸(*e*)], so that the distribution of MQW satellites along the truncation rods can be clearly observed.

In our previous work (Meduňa *et al.*, 2014 ▸) we retrieved a series of 3D RSMs from particular places on a microcrystal using a synchrotron radiation nanobeam at the ERSF. The scanning X-ray nanodiffraction technique using a beam focused down to 0.3 × 0.5 µm was employed in order to map the periodic superlattice on top of isolated individual faceted microcrystals. The process of scanning the diffraction signal over the sample surface also allowed the localization of a particular microcrystal isolated from neighbouring structures with a micromanipulator inside a scanning electron microscope before the scattering experiment. The RSMs were collected with a 2D MAXIPIX detector and, since a well collimated and intense synchrotron beam was available, the 3D RSMs were built directly from the 2D detector images recorded during rocking scans, as typically done in other reports (Etzelstorfer *et al.*, 2014 ▸). Technically, the experiment was realized by scanning the sample on the piezo-stage along the substrate surface and recording the intensity for each point within the *xy* surface plane. Thus, for each pixel on the 2D detector we get a real-space *xy* surface map of diffracted intensity. The surface scans are then repeated for different θ_i_ incidence angles, so that the 3D RSM can be built for every measured *xy* surface point. As a result, we get a detailed five-dimensional map (three dimensions in reciprocal space and two in real space) across the microstructure (Falub *et al.*, 2013 ▸; Meduňa *et al.*, 2014 ▸, 2016 ▸; Meduňa, Isa *et al.*, 2018 ▸). Since the beam is much smaller than the probed microcrystal, different parts (facets or edges) on top can be selected in order to view the 3D RSM at that place. Geometric limitations on beamline ID01 only allowed us to measure the asymmetric (115) RSM, which is however restricted to a fixed azimuthal orientation, especially in the nanobeam regime. On the other hand the technique using the RT, described previously, requires azimuthal rotations to be available for symmetric diffractions. The characteristic faceted shape of the SiGe microcrystal allows us to compare the (115) and (004) RSMs at least qualitatively, as seen below.

**Figure 10:**
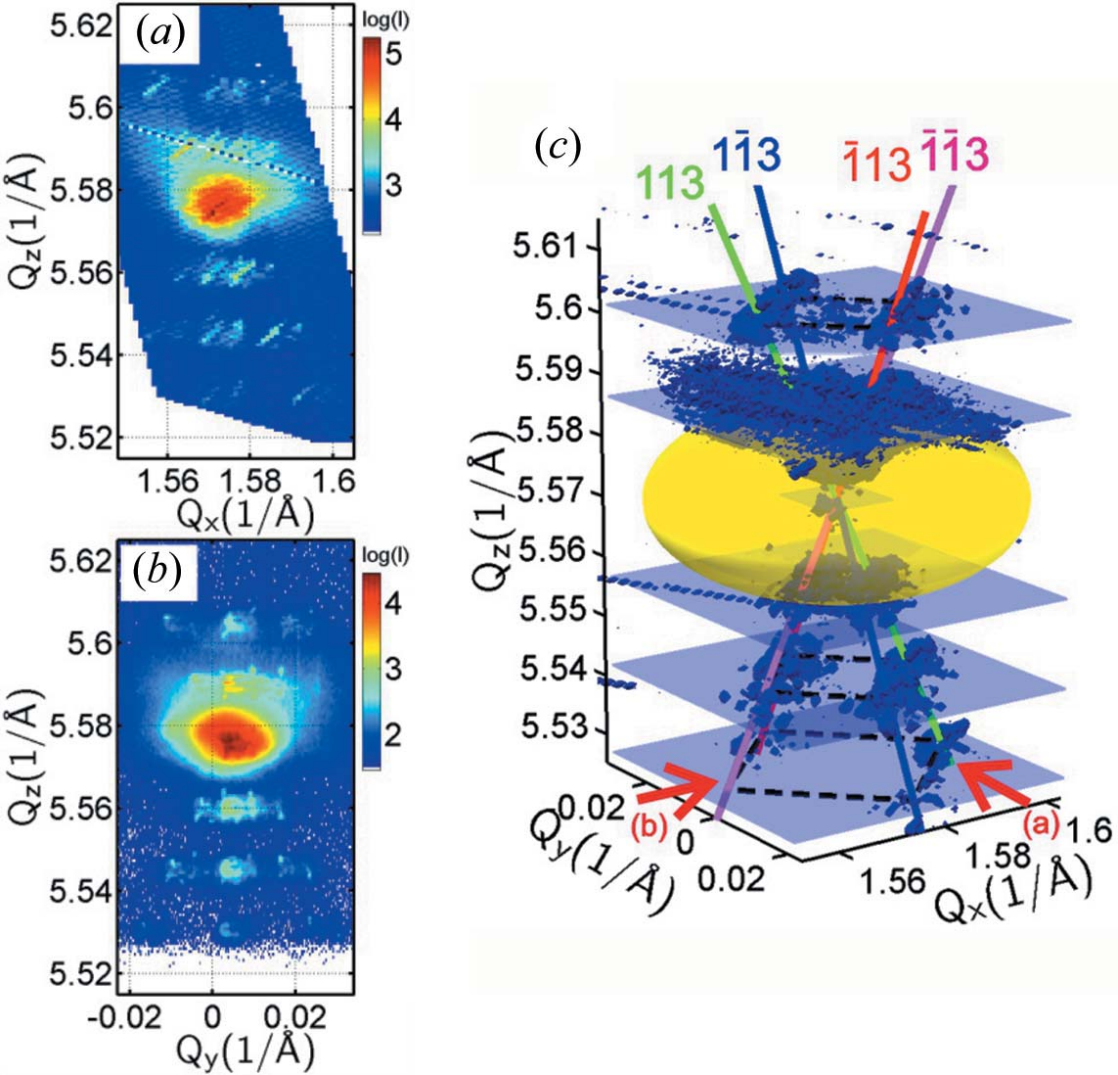
Asymmetric (115) RSMs of the individual SiGe microcrystal containing MQWs recorded during a nanodiffraction experiment at the ESRF and projected at two perpendicular directions of the incidence plane for (*a*) φ = 0° and (*b*) φ = 90°. (*c*) A perspective view of the 3D asymmetric (115) RSM reconstructed from a series of scans with a 2D detector using a nanofocused synchrotron beam placed in the middle of the microcrystal. The red arrows show projection views of the maps in panels (*a*) and (*b*).

For comparison, we show an example of such a 3D RSM in Fig. 10 ▸(*c*). If the nanobeam hits only one facet at the surface of an individual microcrystal, just the single corresponding truncation rod would be detected (Meduňa *et al.*, 2014 ▸). In this case the nanobeam 3D RSM would be quite different from the 3D RSM retrieved from a large beam placed over many thousands of microcrystals. Thus the RSM used for comparison was recorded very close to the central position of a microcrystal so that all four facets were at least partially illuminated by the focused beam. Projections (*Q*_*x*_ *Q*_*z*_) and (*Q*_*y*_ *Q*_*z*_) of this RSM for the azimuths indicated by red arrows are depicted in Figs. 10 ▸(*a*) and 10 ▸(*b*). The central part around the GeSi main peak was eliminated again and replaced by a yellow ellipsoid in order to have a clear overview of the distribution of MQW satellites. We again observe four (113) truncation rods, depicted by red, green, blue and magenta lines.

We see in fact that the 3D RSMs shown in Figs. 9 ▸(*e*) and 10 ▸(*c*) are nearly the same, but for the latter synchrotron radiation was required. The difference in these RSMs is that the former was collected from a large array of microcrystals over an area of about 10 mm^2^ and the latter was obtained locally in the centre of a particular microcrystal with the size of the beam spot being around 0.2 µm^2^. Because of such beam localization and the scanning diffraction technique of the synchrotron experiment, an additional advantage is that we can tune and enhance the intensity of a truncation rod selected from one of the four, depending on which facet is hit by the focused nanobeam. This was the main topic of the previous paper (Meduňa *et al.*, 2014 ▸). Additionally, the shapes of all the peaks are modulated by a resolution function in the form of a disc due to the Fresnel zone plate used for beam focusing [see Figs. 10 ▸(*a*) and 10 ▸(*b*)], which influences the resolution of the experiment as described by Meduňa *et al.* (2014 ▸). The resolution of the 3D RSMs reconstructed by the RT is in principle limited by the pixel size of the 2D detector, which is relatively large. One can also expect, however, that the final resolution of the 3D RSM will be more complex because the RT involves several FFT processing steps, spatial recalibration and frequency filtering. Nevertheless, we can be sure that the 3D RSMs depicted in Figs. 9 ▸(*e*) and 10 ▸(*c*) are comparable and provide almost the same basic overview of the intensity distribution in reciprocal space.

## 6. GaAs/Ge microcrystals

Another complex sample in which we can demonstrate the effect of 3D RSM reconstruction from 2D RSMs obtained at different azimuths and processed using the inverse RT is an array of GaAs/Ge microcrystals grown on a patterned Si pillar substrate with a 6° offcut. These substrate pillars are 15 µm wide and separated by 4 µm wide trenches. First a 2 µm thick Ge mesa layer was epitaxially grown by LEPECVD, followed by 4 µm tall GaAs crystals deposited by metal–organic vapour-phase epitaxy (Taboada *et al.*, 2014 ▸). In our previous work (Taboada *et al.*, 2016 ▸), we have already studied the structural properties, such as the crystal facet morphology and the strain status, using high-resolution X-ray diffraction with a typical laboratory setup. The diffractometer with Cu *K*α_1_ radiation was equipped with a four-bounce Ge(220) crystal monochromator and a two-bounce Ge(220) analyser crystal, similarly to this work as described in Section 2. In the current work we demonstrate reciprocal-space mapping in three dimensions, reconstructed from azimuthal scans.

RSMs for diffraction peak analysis of two perpendicular azimuths can only provide limited spatial information, such that the identification of the individual diffraction peaks can sometimes be very difficult. Realizing the RT can help to identify the origin of individual diffraction peaks much better. On the other hand, the accuracy in the determination of the diffraction peaks achieved by the ultrahigh-resolution RSMs is still usually much better if the peaks are spatially well resolved and identified in reciprocal space.

**Figure 11:**
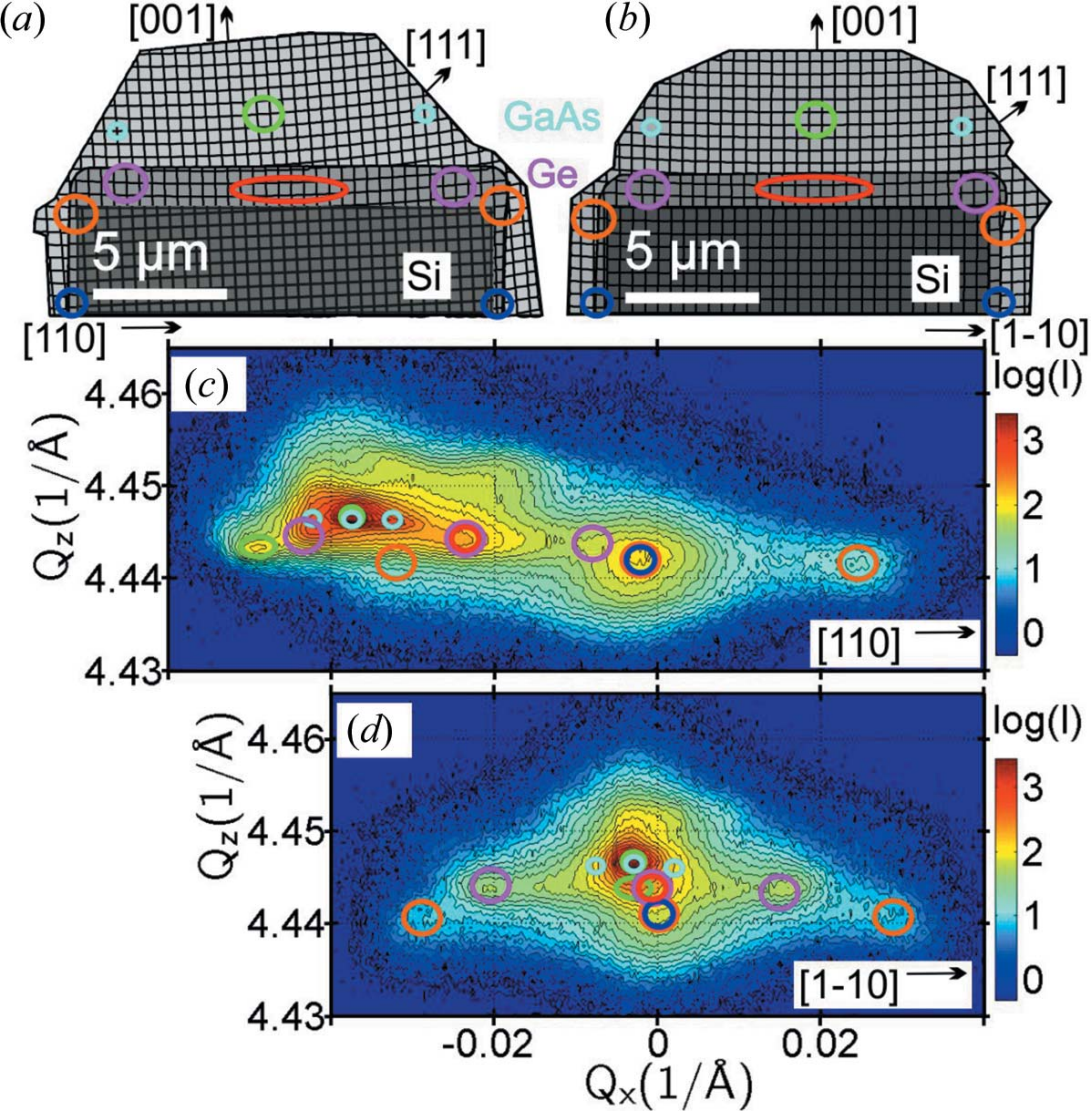
(*a*), (*b*) Sketches showing the microcrystal profile with its structure and the most likely positions of the material responsible for particular diffraction peaks, (*a*) parallel and (*b*) perpendicular to the miscut. (*c*), (*d*) Symmetric high-resolution (004) RSMs recorded under two perpendicular azimuths, (*c*) 0° parallel to the miscut and (*d*) 90° perpendicular to the miscut. The circles around the peaks in the RSMs are roughly associated with the positions in the sketches in panels (*a*) and (*b*) marked by circles of the same colour.

The structure of these GaAs/Ge microcrystals is additionally complicated by thermal strain induced by cooling from the growth temperature down to room temperature. The different thermal expansion coefficients for Si, Ge and GaAs (Si 2.6 × 10^−6^ K^−1^, Ge 5.9 × 10^−6^ K^−1^ and GaAs 5.6 × 10^−6^ K^−1^; Yang *et al.*, 2003 ▸) result in crystal structure bending responsible for the broadening or even splitting of diffraction peaks into two or three maxima (Rozbořil *et al.*, 2016 ▸; Meduňa *et al.*, 2016 ▸). Thus it is useful to consider different parts of the microcrystal lattice to be tilted differently. This was previously confirmed by finite element method calculations of the strain (Taboada *et al.*, 2016 ▸). Moreover, the crystal structure tilt is additionally influenced by the large substrate offcut. A model of the microcrystal structure derived from SEM images including the lattice misorientation is depicted in Figs. 11 ▸(*a*) and 11 ▸(*b*) for two perpendicular azimuthal views (Taboada *et al.*, 2016 ▸). The positions indicated by coloured circles show from where some of the significant diffraction signals in the measured RSMs most probably come.

In order to analyse the strain inside the GaAs/Ge microcrystals, HRXRD RSMs are required to determine individual (*Q*_*x*_, *Q*_*z*_) peak positions for symmetric and asymmetric reciprocal-lattice points. The symmetric diffraction RSM is sufficient for the determination of the crystal structure tilt and an asymmetric one is needed to deduce vertical and horizontal strain. Ultrahigh-resolution (004) RSMs obtained for two perpendicular azimuths of a GaAs/Ge microcrystal sample are shown in Figs. 11 ▸(*c*) and 11 ▸(*d*). We focus only on the peak distribution for symmetric diffraction in this work, which is readily applicable to the RT.

We note that using the RT for asymmetric diffraction points is much more complex, since the geometry of asymmetric diffraction in coplanar scattering does not allow easy azimuthal rotation around φ while keeping the diffraction point close to the scattering plane. The idea of the RT would be applicable to asymmetric reciprocal-lattice points only if a very strange scattering setup was realized. In this case, the sample would have to be mounted on a φ rotation stage in such a way that the (*h*, *k*, *l*) reciprocal-lattice vector pointing to the measured asymmetric diffraction point was also parallel with the φ rotation axis. Since this rotation axis is usually very far from the surface normal of the sample, symmetric and asymmetric diffraction RSMs have to be collected independently, each with separate alignment.

Thus, while the symmetric diffraction RSMs used with the RT can be utilized to identify peaks in 3D reciprocal space, lateral lattice parameters are better determined from ordinary asymmetric HRXRD RSMs. At first glance, these RSMs in Figs. 11 ▸(*c*) and 11 ▸(*d*) exhibit quite a large number of satellite peaks around the main GaAs (004) diffraction point. Their distribution is significantly different for the two azimuths, making the correct peak assignment potentially quite complex. Although finding the peak origin is still possible with only the two azimuthal projections, some kind of 3D imagination is always necessary. The application of the RT combining the RSMs from many azimuths can help significantly in this case, although the spatial resolution used with the 2D HyPix detector is much lower than for the ultrahigh-resolution RSMs of Figs. 11 ▸(*c*) and 11 ▸(*d*) acquired with the Bartels monochromator and analyser crystal.

The individual satellite diffraction maxima are marked by coloured circles corresponding to the expected positions in the crystal from which the signal may come [Figs. 11 ▸(*a*) and 11 ▸(*b*)]. The signals at the very lowest *Q*_*z*_ (blue and orange circles) stem from the microcrystal Ge walls/trenches, those at the mid-range *Q*_*z*_ positions (red and magenta circles) typically originate from the Ge layer, and the highest *Q*_*z*_ positions (small green and cyan circles) can be assigned to the GaAs microcrystals on top, including possible bending at the microcrystal borders. The lattice bending within the GaAs or Ge layer is manifested by the peak splitting into a central maximum and side maxima for each layer. In Fig. 11 ▸(*c*), the peaks from the different materials have varying tilts due to the substrate offcut lying within this [110] azimuth. The RSM in Fig. 11 ▸(*d*) is nearly symmetric for all peaks since the crystal offcut is perpendicular to the scattering plane in this case.

**Figure 12:**
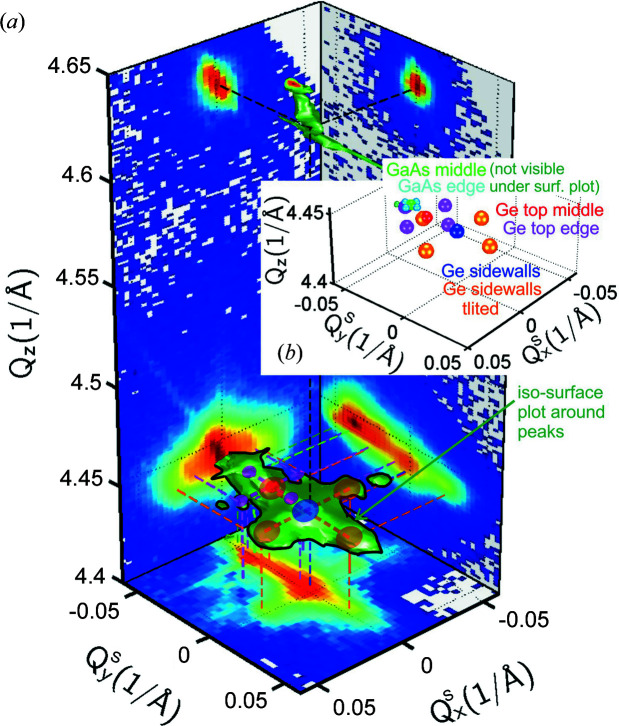
(*a*) A perspective view of the 3D surface plot around the symmetric (004) diffraction peaks of the GaAs/Ge/Si sample in reciprocal space, reconstructed using the Radon transform from *Q*_*x*_ *Q*_*z*_ RSMs recorded under different azimuths φ. The projections on the 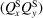 , 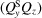 and 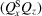 planes show the intensity from different kinds of maxima integrated along *Q*_*z*_, 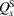 and 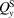 , respectively. (*b*) A 3D sketch of the different intensity maxima originating from various strained parts of the GaAs/Ge microcrystal. Green and cyan: top GaAs peaks in the middle and on the edge. Red and magenta: top Ge layer peaks in the middle and on the edge. Blue and orange: peaks from Ge in walls or trenches at the sides. A solid black line outlining the perspective isolevel (green) surface plot in panel (*a*) has been drawn to illustrate better the shape of the 3D intensity signal.

In order to obtain a better overview of the diffraction peak distribution in 3D reciprocal space, we collected a series of low-resolution 2D RSMs under 90 different azimuths using the same 2D HyPix detector as in the previous sections of this paper. For each *Q*_*z*_ position we built a sinogram similar to the ones in Figs. 4 ▸ and 5 ▸, and using the procedure described in Section 4 we applied the RT. The reconstructed 3D RSM in perspective view is shown in Fig. 12 ▸(*a*), wherein we observe the distribution of some of the satellites of the diffraction peaks.

The peaks are arranged in predominantly fourfold structures with one central maximum surrounded by four side maxima caused by crystal structure bending towards the microcrystal edges, resulting in a concave bowl shape. From the 3D intensity distribution we observe that the diffraction peaks are cross-shaped and oriented along the 〈110〉 edges of the microcrystal [see also the sketch in Fig. 12 ▸(*b*)]. Such a cross is clearly observed first for the Ge deposited on the side walls of the Si pillar and in the trenches in between (blue and orange spheres). A second smaller cross is at the limit of visibility for the Ge interlayer as well, slightly shifted by the tilt along the [110] direction due to the offcut (red and magenta spheres). Most probably the last cross, unresolved in the 3D RSM of Fig. 12 ▸(*a*) due to the low resolution of the collected 2D (*Q*_*x*_ *Q*_*z*_) RSMs at different azimuths, can be deduced in the ultrahigh-resolution RSM projections of Fig. 11 ▸(*c*) at the GaAs position indicated by green and cyan circles. This one is very small but intense, being associated with the GaAs structure. The GaAs microcrystal dominates the GaAs (004) peak and some broadening with wings is evident in Fig. 11 ▸(*c*). Since the top shape of the GaAs microcrystal is no longer fourfold symmetric due to the significant influence of the offcut on the top facets, the side maxima of GaAs are not well resolved even in the perpendicular azimuth of the ultrahigh-resolution RSM in Fig. 11 ▸(*d*). In order to resolve the GaAs peak better, an ultrahigh-resolution 3D RSM, built from higher pixel resolution in 2D RSMs, would be necessary. However, in order to increase also the resolution in the horizontal (*Q*_*x*_ *Q*_*y*_) plane of the 3D RSM, larger numbers of azimuthal positions would be required for the RT process as well.

A complete overview of the spatial distribution of the cross-shaped diffraction peaks is shown as coloured spheres in the 3D 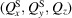 coordinate system in Fig. 12 ▸(*b*), and this also helps to explain the iso-intensity surface plot of Fig. 12 ▸(*a*). The peak distribution, at least the largest cross-shaped peaks, is visible in the 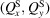 projection obtained as a sum of intensities along *Q*_*z*_. The other two 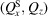 and 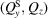 projections are built similarly and provide the same picture as in Figs. 11 ▸(*c*) and 11 ▸(*d*) but with the limited resolution of the RT, mainly as a result of the large detector pixel acceptance, yet still show excellent correspondence with the HRXRD data. However, if a much larger spatial resolution is available in the RSMs, the RT processing has the potential to provide a very detailed 3D spatial distribution of all diffraction peaks. Unfortunately, if an analyser crystal and point-by-point scanning have to be used, the measurement time becomes un­acceptably long.

## 7. Conclusions

We have presented here a new processing technique to build 3D X-ray diffraction reciprocal-space maps around symmetric diffraction points using a 2D pixel detector in linear mode with a standard laboratory setup and linear slits. The intensity distribution in 3D reciprocal space was reconstructed from series of RSMs recorded under different azimuthal sample orientations, using the well known Radon transform processing typically applied for medical and materials imaging in real space. The Radon transform in reciprocal space has been demonstrated for three different examples. The method has been described in detail for a relatively simple example of elongated SiGe microcrystals for which RSMs showed two sets of fourfold satellite maxima arranged in squares. The reconstruction of the individual maxima works very well here due to their low intensity. On the other hand, the reconstruction of the main high-intensity diffraction peak fails because the application of the Radon transform is limited by the dynamic range and high noise starts to appear due to necessary frequency filtering. The second, more complex, example consisted of faceted Ge microcrystals containing Ge/SiGe multilayers, where the multilayer satellite peaks are arranged along different spatial directions. Excluding the main high-intensity peak, the reconstructed 3D RSM was qualitatively the same as the one obtained at a synchrotron beamline earlier. The last, most complex, example was a sample comprising GaAs microcrystals, exhibiting a mixture of many spatially distributed diffraction peaks. The application of the Radon transform allowed us to reveal that these maxima form nearly fourfold symmetric cross-shaped features associated with thermal lattice bending inside the layered parts of the microcrystal. These features would also, in principle, be easily observable in a 3D RSM recorded with a highly collimated small-sized synchrotron beam when rocking the incidence angle and using a 2D pixel detector placed far from the sample. However the RT processing of typical RSMs, recorded at a set of different azimuths, is also readily accessible within a standard diffractometer equipped with any linear detector.
